# What bats have to say about speech and language

**DOI:** 10.3758/s13423-016-1060-3

**Published:** 2016-07-01

**Authors:** Sonja C. Vernes

**Affiliations:** 1 Max Planck Institute for Psycholinguistics, Nijmegen, The Netherlands; 2 Donders Institute for Brain, Cognition and Behavior, Nijmegen, The Netherlands

**Keywords:** Bats, Vocal learning, Turn-taking, Language, Language evolution, Speech

## Abstract

Understanding the biological foundations of language is vital to gaining insight into how the capacity for language may have evolved in humans. Animal models can be exploited to learn about the biological underpinnings of shared human traits, and although no other animals display speech or language, a range of behaviors found throughout the animal kingdom are relevant to speech and spoken language. To date, such investigations have been dominated by studies of our closest primate relatives searching for shared traits, or more distantly related species that are sophisticated vocal communicators, like songbirds. Herein I make the case for turning our attention to the Chiropterans, to shed new light on the biological encoding and evolution of human language-relevant traits. Bats employ complex vocalizations to facilitate navigation as well as social interactions, and are exquisitely tuned to acoustic information. Furthermore, bats display behaviors such as vocal learning and vocal turn-taking that are directly pertinent for human spoken language. Emerging technologies are now allowing the study of bat vocal communication, from the behavioral to the neurobiological and molecular level. Although it is clear that no single animal model can reflect the complexity of human language, by comparing such findings across diverse species we can identify the shared biological mechanisms likely to have influenced the evolution of human language.

## Animal models relevant to speech and language

The comparative approach, investigating similar traits across diverse species, has been a driving force in understanding the genetics, physiology, and evolution of complex traits. The study of such traits in experimental animal models has shed light on human-relevant processes involved in both normal development and disease states. Despite their power, applying these approaches to the study of spoken language presents particular difficulties, given that (a) spoken language does not leave direct evidence in the fossil record, making comparisons to our extinct ancestors challenging, and (b) humans are the only extant species with this trait.

Although language is not found in other animals, some aspects of social and vocal animal communication are likely to inform us about how this trait evolved (Fitch, Huber, & Bugnyar, 2010). Vocal learning is an essential component of spoken language, and songbirds have been the dominant model used to study this trait, due to their well-defined learning paradigm, sexual dimorphism of their song, and the ability to breed these animals in captivity (Condro & White, 2014). Songbirds have been a success story, revealing much about the wiring of specific circuits (e.g., the anterior forebrain pathway; Doupe, Solis, Kimpo, & Boettiger, 2004) and genetic components (e.g., *FoxP2*; Haesler et al., 2007) involved in this complex behavior. However similar levels of success have not been achieved outside avian model systems, and thus exactly how these mechanisms translate to the mammalian brain still demands investigation. Conversely, primate studies have revealed complex nonverbal communication abilities (e.g., communication via sign language or visual referential systems), but despite intensive training, primates have never been able to acquire language abilities greater than those of a human toddler, or indeed any capacity for speech (Fitch, 2000).

Given spoken language’s exclusivity to humans, no single animal model is going to provide all of the answers regarding how it is encoded or how it evolved. Rather, we need to seek clues from a range of phylogenetically diverse species with language-relevant traits, exploiting both observational studies and, where possible, animal models amenable to laboratory studies. Only by integrating this information across species will we be able to build a picture of the essential components that would allow an organism to develop and employ spoken language.

## The promise of chiroptera

One order of animals that are particularly promising for the study of language-relevant traits is Chiroptera (bats). Bats arose ~64 million years ago and are the second-largest group of mammals (behind rodents), with 18 families representing extensive evolutionary and functional diversity (Teeling et al., 2005). As mammals, bats share brain structures such as a six-layered cortex and hippocampus with humans and other mammals, which is a considerable advantage when interrogating the roles of these regions (and the associated circuitry) in complex, spoken-language-relevant tasks.

Bats have developed sophisticated vocal systems for navigation and communication, and many species rely on echolocation to navigate their environment and hunt prey. Most echolocating bats produce calls from the larynx (see Au & Suthers, 2014, for a comprehensive review of bat laryngeal control and biosonar production) and use the returning echoes to detect the structure of their environment. This means that echolocating bats are highly skilled at producing precise, rapid vocalizations, and their auditory systems and neurobiology are exquisitely tuned to acoustic information—important factors also required for the development of complex vocal communication. Echolocation has been well-studied in bats, and thus the functionality of different call types (Jones & Teeling, 2006), neurobiological mechanisms (Moss & Sinha, 2003), and genetic evolution (Parker et al., 2013) are becoming well understood (Jones, Teeling, & Rossiter, 2013).

Vocalizations (sonic and/or ultrasonic) are also heavily employed by bats to facilitate social communication (Pfalzer & Kusch, 2003). Most bats are highly social, living in groups (often fission–fusion groups) that may range from a small roost of a few animals to large colonies with millions of inhabitants. Social communication in these environments has been found in the context of allogrooming, mother–pup interactions, group contact calls, foraging calls, mate attraction, and territorial defense (Behr & von Helversen, 2004; Kerth, 2008; Pfalzer & Kusch, 2003; Wilkinson, Carter, Bohn, & Adams, 2016). However the diversity of bat species and their frequently inaccessible habitats, ultrasonic form of some vocalizations, and abundance of call types, mean that much is still to be understood about the range and function of social vocalizations.

I posit that given their complex vocalizations, biological specification for vocal control/auditory processing, and diverse family tree, bats represent a highly appealing yet currently understudied system to investigate the biological basis and evolution of spoken language in a mammalian system. Herein I discuss two facets of vocal communication that have relevance to human spoken language and for which bats show great promise: vocal learning and vocal turn-taking.

## Learned vocal communication

Vocal learning can be classified into vocal production learning (sensory–motor learning) and contextual learning (the usage or comprehension of vocalizations; Janik & Slater, 2000). Vocal production learning (VPL) is the ability to modify vocalizations in response to interactions with conspecifics, and allows human infants to advance from producing incoherent babbling to a meaningful vocal lexicon. VPL is a central component needed for the evolution of human speech and involves the integration of information across modalities, including auditory perception, memory, and motor production. To learn a vocalization, relevant sounds produced by conspecifics must be recognized and this vocal template must be remembered. Then a motor program attempting to mimic these sound(s) must be planned and enacted. The output of this motor program must be compared against the template to determine match/mismatch, and if necessary, adjustments must be made until the output matches the template. Social interactions between conspecifics may also reinforce the selection of the appropriate outputs (West & King, 1988).

There is strong evidence for VPL in only a handful of nonhuman animals, including some birds (songbirds, parrots, and hummingbirds), pinnipeds (seals), cetaceans (whales), elephants, and some bats (Janik & Slater, 1997; Knörnschild, 2014; Petkov & Jarvis, 2012). However it has been hypothesized that the lack of evidence in a larger number of species may be due to insufficient study, and indeed, more wide-ranging investigations may point to a continuum of vocal-learning abilities rather than a simple classification of animals as vocal learners versus nonlearners (Petkov & Jarvis, 2012). Bats present a highly promising system through which to study behavioral and biological aspects of vocal learning. Despite their extensive speciation and diverse vocal and echolocation mechanisms (e.g., constant-frequency vs. frequency-modulated calls), bats from across the Chiropteran family tree display evidence for vocal learning (Knörnschild, 2014).

### Vocal learning in phyllostomidae—open-ended learning

Direct experimental evidence for VPL in bats first came from social communication calls produced by the pale spear-nosed bat (*Phyllostomus discolor*) in the Phyllostomidae family. *P. discolor* pups emit isolation calls in response to frequency-modulated (FM) maternal “directive” calls that are used for mother–pup identification/reunion (Rother & Schmidt, 1985). Naturally reared pups modify their isolation calls to adopt the FM properties of their mother’s call (Esser & Schmidt, 1989), and hand-reared pups adapt to the FM properties of digital directive calls, demonstrating their ability to learn from a conspecific template (Esser, 1994). It is not clear whether *P. discolor* bats have a critical period (like zebra finch songbirds) or are able to continue learning new vocalizations into adulthood (like parrots, whales, or dolphins). However *Phyllostomus hastatus*, one of the species most closely related to *P. discolor*, is an open-ended learner, able to learn vocalizations into adulthood. *P. hastatus* bats form stable social groups of unrelated females that produce a signature group contact call, known as a *screech call* (Boughman, 1998). When animals join a new group, both the existing “residents” and new members (both juvenile and adult) adapt their call characteristics to establish a new group signature call (Boughman, 1998). Since *P. hastatus* is an open-ended learner, it is likely that *P. discolor* bats will also be able to continue learning vocalizations into adulthood, but controlled experimental paradigms directly proving this will be essential.

### Vocal learning in emballonuridae—babbling and learned song

The greater sac-winged bat (*Saccopteryx bilineata*) in the Emballonuridae family produces FM echolocation calls and a rich repertoire of social communication calls, including isolation calls, courtship songs, and territorial songs (Behr & von Helversen, 2004). Their social vocalizations have been found to contain individual and group signature information, and their echolocation calls contain individual and sex signatures (Eckenweber & Knörnschild, 2013; Knörnschild, Jung, Nagy, Metz, & Kalko, 2012; Knörnschild, Nagy, Metz, Mayer, & von Helversen, 2012). *S. bilineata* juveniles learn territorial songs (containing individual and group signatures) that the males use to defend their home roost against invasion from adult males (Eckenweber & Knörnschild, 2013; Knörnschild, Nagy, Metz, Mayer, & von Helversen, 2010). Despite this sexual dimorphism, both sexes produce all possible adult call types as pups, and during ontogeny these calls are flexibly practiced in a fashion reminiscent of songbird vocal learning and human infant babbling (Knörnschild, Behr, & von Helversen, 2006; Knörnschild et al., 2010). This species offers exciting opportunities for the study of spoken language-relevant traits, given the presence of both learned vocalizations and babbling behavior.

### Vocal learning in pteropodidae—juvenile vocal development

The Egyptian fruit bat (*Rousettus aegyptiacus*) is a large Old World megabat from the family Pteropodidae. Old World fruit bats do not use laryngeal echolocation; instead, genus *Rousettus* have developed a method for echolocation that involves producing tongue clicks (Yovel, Geva-Sagiv, & Ulanovsky, 2011). Recently, *R. aegyptiacus* provided evidence of vocal learning during bat juvenile development. Prat, Taub, and Yovel (2015) undertook the daunting task of documenting the complex vocal ontogeny of *R. aegyptiacus* over a 9-month period, producing a dataset of >1 million calls (Prat et al., 2015). By observing animals raised in the presence of adults as compared to isolated animals that only interacted with other isolate juveniles, they were able to demonstrate that exposure to adult vocalizations influenced the development of the *R. aegyptiacus* vocal repertoire (Prat et al., 2015). Isolate animals maintained a significantly different call from those of normally reared adults, and moreover, they could be induced to shift the frequency of their vocalizations by exposure to playback of calls rarely made by normal adult *R. aegyptiacus* (Prat et al., 2015). This work suggests that some form of vocal learning (either production or contextual) is required for the normal development of *R. aegyptiacus* and illustrates the experimental promise of bats for characterizing vocal-learning behavior. In the future, it will be of great interest to extend these paradigms to show whether these bats are capable of learning novel sounds never normally produced by adult bats.

In summary, evidence is accumulating that bats from multiple species are capable of vocal learning. In fact, the species for which vocal learning has been demonstrated are found spread throughout the Chiropteran phylogenetic tree, with promising or confirmed vocal learners identified in around half of the 18 families of bats, including across the two major bat suborders, Yinpterochiroptera and Yangochiroptera (Knörnschild, 2014; Prat et al., 2015). This suggests that with further studies we may find that vocal learning is a general, or at least highly prevalent, feature of bat behavior, providing a rich framework in which to investigate this spoken-language-relevant trait. Given that some vocal-learning bat species can be housed in laboratory colonies (e.g., *P. discolor* and *R. aegyptiacus*), this affords new opportunities to directly investigate the neurobiological and genetic models proposed from avian studies (Bouchard & Brainard, 2013; Brainard & Doupe, 2013; Scharff & Petri, 2011; White, 2010) in a mammalian brain. Such comparative studies will be an important step in finding common mechanisms underlying the evolution of vocal learning in humans.

## Taking turns during vocal communication

Human language is exceedingly diverse, and few (hotly debated) universal properties have been proposed that are shared by all known languages (For examples, see Greenberg, 1963, and Evans & Levinson, 2009, together with the latter’s numerous published responses.) One strong candidate for a language universal is the property of vocal turn-taking. The majority of language use involves conversational interactions between two or more parties and relies on the rapid switching of turns between agents (speakers or signers). In humans, the timing of these turns is highly similar not only within a language, but across all languages studied to date, including signed languages (Levinson, 2016; Stivers et al., 2009). Given the rapid time scale at which turn-taking occurs (~200 ms), as compared to the relatively slow act of speech production planning (>600 ms), successful turn-taking must involve intensive cognitive multitasking, in order to simultaneously comprehend meaning and plan the production of a rapid yet coherent response (Levinson, 2016). Thus, turn-taking is a demanding, yet seemingly common, feature of human language that may have deep evolutionary roots.

### Animal turn-taking behavior

Humans are not the only animals that employ turn-taking during communication, supporting these putative deep roots (Yoshida & Okanoya, 2005). Turn-taking is observed throughout the animal kingdom, and is hypothesized to subserve functions including mate identification/reunion, joint territory defense, and pair bonding. In animal communication, turn-taking has been described as “alternating signal transmission between participants, with defined reply latency” (Yoshida & Okanoya, 2005, p. 154) and can involve dueting (reciprocal exchange between male–female pairs) or antiphonal vocalizations (alternating call and response between two or more animals). Such vocal turn-taking is found in both vocal-learning and -nonlearning animals, and in many species it occurs soon after birth, suggesting a mechanism that is at least partially innate. Turn-taking is well-studied in birds, where dueting and antiphony are widely found (Dahlin, Benedict, & Hauber, 2014; Henry, Craig, Lemasson, & Hausberger, 2015). Primates also display vocal turn-taking, with evidence across prosimians, monkeys, and lesser apes (Chow, Mitchell, & Miller, 2015; Lemasson et al., 2011; Mendez-Cardenas & Zimmermann, 2009; Snowdon & Cleveland, 1984).

### Turn-taking in bats

Only a small number of studies have explored the possibility of turn-taking in bats; however, there is good evidence that a number of bat species produce antiphonal (call and response) vocalizations. These antiphonal calls have been hypothesized to act as contact calls facilitating individual identification, group cohesion, and/or group territorial defense—similar to what is seen in birds and primates.

Bats from four families (Molossidae, Vespertilionidae, Phyllostomidae, and Emballonuridae) are known to perform a vocal call and response during mother–pup reunions (Balcombe, 1990; Balcombe & Mccracken, 1992; deFanis & Jones, 1996; Esser & Schmidt, 1989; Knörnschild & von Helversen, 2008). Upon returning to a roost, mothers that have been independently foraging call to their offspring to locate them. Pups respond to these “maternal directive calls” with isolation calls that contain individual recognition cues to facilitate further call exchange, guiding the mother to the pup (Knörnschild & von Helversen, 2008). Thus, coordinated control of the call and response (antiphonal calling) between mother and pup is essential for reunion of the pair and the survival of the offspring. This presents a promising area for the study of vocal turn-taking in bats, where it will be important to determine whether these calls meet the temporal criteria given for vocal turn-taking (i.e., a call and response with defined latencies; Yoshida & Okanoya, 2005).

Vocal turn-taking has been found in vampire bats (within the Phyllostomidae family), highly social bats that perform allogrooming and reciprocal food sharing (Carter, Skowronski, Faure, & Fenton, 2008). Adult white-winged vampire bats (*Diaemus youngi*) produce a specific duet-like FM social call when feeding, leaving roosts, or when separated over long distances. *D. youngi* produce these FM social calls within a defined latency, most often 300–350 ms after hearing a conspecific call (Carter et al., 2008). Most of these antiphonal interactions involved a single call and response; however, up to five alternating calls between bats could be observed. These bats also performed antiphonal vocalizations more often when they were physically isolated and could only hear conspecific calling, supporting the vocalizations’ role in long-distance recognition and group cohesion (Carter et al., 2008).

Although it is likely that turn-taking arose due to multiple convergent evolution events across taxa, understanding how this evolved in such distant species as bats and primates might give us clues as to how vocal turn-taking arose in humans. Shared genetic factors have been shown to underlie convergently evolved traits across disparate taxa. A clear example of this is the selection of genes related to hearing (such as *prestin*) in echolocation in both bats and cetaceans (Li, Liu, Shi, & Zhang, 2010; Shen, Liang, Li, Murphy, & Zhang, 2012). Thus, studying examples of antiphonal behavior across bats and other species will reveal underlying biological mechanisms for this intriguing spoken-language-relevant trait, and may point to shared origins with human turn-taking.

## Neurobiology, genetics, and the future

Herein I have discussed evidence that bats present exciting new models with the potential to illuminate the biological underpinnings of traits relevant to language. Clearly there is an urgent need for further ethological work to better define the range and functions of bat vocal behavior. This would allow comparisons across diverse bat species, but importantly, also across taxa including songbirds, primates, and humans. However, it is important not to stop at the behavioral level, but also to make coordinated efforts to understand the neurobiological and genetic mechanisms that build a brain capable of these complex behaviors. To date, such studies have largely been restricted to humans and birds. Advances in genetics and neurobiology are now making it possible to investigate how these traits are encoded in bats, bridging the evolutionary gap between findings in human and avian systems.

### Neurobiology

Studying the neurobiology of language-relevant traits in bats has the benefit of directly investigating a mammalian brain, and a range of techniques are now possible to accurately measure brain structure and activity. Magnetic resonance imaging (MRI) has been used in bats to demonstrate brain architecture and cochlea specializations related to echolocation (Hsiao, Jen, & Wu, 2015; Hu, Li, Gu, Lei, & Zhang, 2006), and functional MRI has shown cortical activation patterns in bats following auditory stimuli (Kamada, Pekar, & Kanwal, 1999). Most recently, wireless electrophysiology has allowed single-cell recording of freely behaving bats, revealing unprecedented insight into how bat brains encode three-dimensional information while in flight (Yartsev & Ulanovsky, 2013; Yartsev, Witter, & Ulanovsky, 2011). The neurobiological mechanisms underlying traits like vocal learning have been extensively studied in the avian brain, and there is now a both a clear need and the technical competency for comparable experiments to be carried out in mammalian systems such as the bat. Conserved brain structure across mammals will allow us to use findings from bats to make direct parallels with humans when considering the role of cortical networks in vocal communicative behavior. Such comparative work will be central to identifying common evolutionary themes underlying these complex traits.

### Genetics

Rapid advances are also being made at the molecular level (e.g., transcriptomes and genomes) and in our ability to perform genetic manipulations. To date, sequence data from ten bats have been released, giving some intriguing clues into the evolution of echolocation, flight, and immunity (Li, Wang, Rossiter, Jones, & Zhang, 2007; Parker et al., 2013; Zhang et al., 2013). However, to understand the evolution of social communication, further Chiropteran genomes coupled to in-depth behavioral analysis will be essential, as has been demonstrated by the valuable work of the Avian Phylogenomics Project (Pfenning et al., 2014; Whitney et al., 2014). Overlaid with this, transcriptomic (gene expression) data will give us direct insight into how molecular mechanisms function in the real-time processing of these traits. We recently identified functional gene networks via transcriptomics in the *P. discolor* brain (Rodenas-Cuadrado, Chen, Wiegrebe, Firzlaff, & Vernes, 2015), demonstrating the feasibility of interrogating the molecular pathways underlying complex traits in the bat brain. Combining such cutting-edge molecular and neurobiological techniques in bats with ethological studies will reveal new insight into the encoding and function of circuits involved in complex communicative behavior in the mammalian brain.

### The future

It is clear that no single animal or animal behavior can be used to accurately model language. Thus, it is crucial that we explore a wide range of language-relevant traits across diverse species. Bats have enormous potential to contribute to this field, opening up new experimental avenues to understand how a mammal develops these traits, and how this development compares with that of more distantly related species, like birds. Taking an integrative approach that considers findings from birds, to bats, and beyond will provide essential insights into the biological encoding of complex communication systems, a key step toward understanding the evolution of human speech and language.
